# Growth Factors, PI3K/AKT/mTOR and MAPK Signaling Pathways in Colorectal Cancer Pathogenesis: Where Are We Now?

**DOI:** 10.3390/ijms221910260

**Published:** 2021-09-23

**Authors:** Constantin Stefani, Daniela Miricescu, Iulia-Ioana Stanescu-Spinu, Remus Iulian Nica, Maria Greabu, Alexandra Ripszky Totan, Mariana Jinga

**Affiliations:** 1 Department of Family Medicine and Clinical Base, ‘‘Dr. Carol Davila’ Central Military Emergency University Hospital, 051075 Bucharest, Romania; constantin.stefani@umfcd.ro; 2 Department of Biochemistry, Faculty of Dental Medicine, Carol Davila University of Medicine and Pharmacy, 8 Eroii Sanitari Blvd, 050474 Bucharest, Romania; iulia.stanescu@umfcd.ro (I.-I.S.-S.); alexandra.totan@umfcd.ro (A.R.T.); 3 Surgery 2, ‘Dr. Carol Davila’ Central Military Emergency University Hospital, 051075 Bucharest, Romania; nica.remus@yahoo.com; 4 Department of Gastroenterology, ‘Dr. Carol Davila’ Central Military Emergency University Hospital, 051075 Bucharest, Romania; mariana.jinga@umfcd.ro

**Keywords:** colorectal cancer, growth factors, PI3K/AKT/mTOR, MAPK, growth factor inhibitors

## Abstract

Colorectal cancer (CRC) is a predominant malignancy worldwide, being the fourth most common cause of mortality and morbidity. The CRC incidence in adolescents, young adults, and adult populations is increasing every year. In the pathogenesis of CRC, various factors are involved including diet, sedentary life, smoking, excessive alcohol consumption, obesity, gut microbiota, diabetes, and genetic mutations. The CRC tumor microenvironment (TME) involves the complex cooperation between tumoral cells with stroma, immune, and endothelial cells. Cytokines and several growth factors (GFs) will sustain CRC cell proliferation, survival, motility, and invasion. Epidermal growth factor receptor (EGFR), Insulin-like growth factor -1 receptor (IGF-1R), and Vascular Endothelial Growth Factor -A (VEGF-A) are overexpressed in various human cancers including CRC. The phosphatidylinositol 3-kinase (PI3K)/protein kinase B (AKT)/mammalian target of rapamycin (mTOR) and all the three major subfamilies of the mitogen-activated protein kinase (MAPK) signaling pathways may be activated by GFs and will further play key roles in CRC development. The main aim of this review is to present the CRC incidence, risk factors, pathogenesis, and the impact of GFs during its development. Moreover, the article describes the relationship between EGF, IGF, VEGF, GFs inhibitors, PI3K/AKT/mTOR-MAPK signaling pathways, and CRC.

## 1. Introduction

If, in 1950, colorectal cancer (CRC) was a rare malignancy, today, it became a predominant form worldwide [1]. After breast, lung, and prostate cancer, CRC is the fourth most common cause of cancer [2] and aggressive malignancy [3]. In the United States, it is the second-leading cause of death [2]. Every year, more than 1.2 million patients are diagnosed with CRC, and more than 600,000 lose the battle with this disease [4]. Worldwide, CRC is the third most common cancer, and the incidence is increasing with age [5,6,7]. In Europe, around 11% of CRC cases are attributed to overweight and obesity, especially visceral fat or abdominal obesity. The epidemiologic studies reported an incidence of 30–70% increased risk of CRC in obese men [8]. The most common CRC subsets are colon, proximal colon, distal colon, and rectum [9]. Since 1994, the CRC incidence in adolescents and young adults under 45 years has been increasing every year [10,11,12,13]. The statistical data published in 2014 revealed that 26% of proximal colon cancers were diagnosed in women younger than 50 years, while 56% of the cases were registered in women aged 80 years and older [14]. Compared with older CRC patients, early-onset CRC is a heterogenous group that is distinct from the clinical, pathologic, and molecular points of view [15]. Therefore, an increased incidence was observed between 49 and 50 years [16]. Kim SE reported that women are more prone to developing right-sided (proximal) colon cancer compared with men. Proximal colon cancer is a more aggressive form versus the left-sided (distal) form [17]. Depending on the mutation origin, CRC carcinomas are classified as sporadic (70%), inherited (5%), and familial (25%). Unfortunately, metastatic CRC (mCRC) is often incurable in most cases, representing 13% of all diagnosed cancers [18,19], with an overall survival rate of 13% [18,20]. Corroborating all the information received from genomic, epigenomic, transcriptomic, and microenvironment levels, CRC has molecular heterogeneity. Moreover, genomic events accumulated during carcinogenesis remain the leaders of cancer progression in the metastatic stage [21]. For early CRC, the 5-year survival rate is ~90%, but this rate decreases to 15% in the case of mCRC [22].

## 2. Risk Factors in CRC

Both environmental and genetic factors are involved in the etiology of CRC [23]. More than 80% of CRC cases are sporadic, as patients do not present a family history [23]. Therefore, the majority of the CRC cases (>90%) can be prevented if they are tested and screened early [24]. Several modifiable risk factors are involved in CRC pathogenesis such as diet, obesity, sedentary life, smoking, and moderate-to-heavy alcohol consumption [25]. Diet plays a pivotal role in CRC development [26,27], the consumption of unhealthy food being a significant factor in CRC development [28]. Moreover, a diet rich in red meats, processed meats, saturated animal fats, spicy foods, refined carbohydrates are associated with increased CRC development [27]. The International Agency for Research on Cancer (WHO-IARC) classified the consumption of processed meat as “carcinogenic to humans”. Several compounds present in red (haem iron) and/or processed meat (nitrates and nitrites) as well as those formed during cooking will react with colorectal mucosa and promote carcinogenesis [29]. Experimental studies, performed on rodent models, reported that red meat haem iron induces lipid oxidation with the formation of 4-hydroxynonenal (HNE) from n-6 fatty acids. Aldehydes’ synthesis is correlated in rats with preneoplastic lesions. In vitro, it has been observed that haem iron and aldehydes can enhance cellular inflammatory processes and cellular permeability, as well as promoting cellular DNA damage [30]. The process of meat cooking can incorporate or develop mutagens and carcinogens, which have been shown to enhance carcinogenesis. During high-temperature or open-flame meat cooking, heterocyclic amines (HCAs) and polycyclic aromatic hydrocarbons (PAHs) are formed. In meat, the most common PAH compound is benzo(a)pyrene. Cytochrome P450 enzymes activate these pro-carcinogens, which will be further converted in several metabolic pathways [31]. Moreover, N-nitroso compounds (NOC) obtained by the interaction between nitrogen oxides or nitrite with secondary amines and N-alkillamides have CRC carcinogenic properties [32]. In addition, the consumption of red meat and other animal products is conducive to trimethylamine N-oxide (TMAO) synthesis, a gut microbiota-derived metabolite of choline and L-carnitine, associated with an increased risk of CRC, cardiovascular disease, and diabetes. The correlation between TMAO and cancer is performed via inflammation, OS, DNA damage, and protein folding disruption [33].

The results from the epidemiologic and experimental studies performed in the last few decades revealed that calcium, fibers, milk, and whole grains decrease the CRC incidence, while red and processed meat increase the risk [26]. While the Western society prefers to eat red and processed meat associated with an increased cancer incidence, the Mediterranean diet is correlated with a decreased cancer incidence [34]. Smoking and a sedentary lifestyle are major risk factors for early-onset CRC [35]. Smoking cigarettes generates more than 7000 toxic chemicals, with at least 70 known carcinogens that can affect the entire human body. Carcinogens from the cigarette smoke (nitrosamines, heterocyclic amines, benzene, and polycyclic aromatic hydrocarbons) directly interact with the colorectal mucosa in two ways—by direct ingestion or through the bloodstream. Overall, smoking has a direct oncogenic effect, being correlated with CRC adenoma [36]. Moreover, passive smoking is an independent risk factor for CRC neoplasia in non- and former smokers [37].

In addition, it seems that physical activity after CRC diagnosis may reduce the risk of mortality by 38% [38]. In 2015, Baena R and co-workers published the results of the epidemiologic studies from EMBASE and PubMed-NCBI, carried out since November 2014, and revealed that obesity increases the risk of CRC by 19%, while regular physical activity reduces this risk by 24%. In addition, fish, fibers, and milk consumption reduce the risk of colon cancer [39]. Among students, the most important factors for CRC development are smoking (90.5%), excessive alcohol consumption (87.4%), family history of cancer (84.2%), and obesity (82.6%) [40]. The results of a prospective study regarding the effect of diet on CRC development were published in 2020. The study was conducted over a period of 4 years (2006–2010) and included men and women aged 40–69 years. The study revealed that consumption of 76 g/d red and processed meat and alcohol consumption increase the risk of CRC, while fibers from bread and breakfast cereals were associated with a decreased risk [41]. Ethanol is metabolized to acetaldehyde by alcohol dehydrogenases (ADH), catalase, or cytochrome P450 2E1. Aldehyde dehydrogenase further oxidases ethanal to acetate, a Group 1 carcinogen for humans. In the stomach and colon, the ethanal level is influenced by gastric colonization, by *Helicobacter pylori*, or by colonic enzymes. Furthermore, alcohol generates reactive oxygen species (ROS), leading to DNA damage and activating signaling pathways involved in inflammation, metastasis, and angiogenesis [42].

Diabetes is another risk factor for CRC [43]. An elevated body weight associated with a sedentary lifestyle plays an important role in CRC pathogenesis [43]. A link between insulin resistance (IR), hyperinsulinemia and cancer, and changes in the expression of insulin receptors and insulin growth factor (IGF) system, including IGF-I, IGF-II, has been observed. When insulin binds to IGF-1 receptor (IGF-1R) with low affinity, cell proliferation is stimulated via phosphatidylinositol 3 kinase (PI3K)/protein kinase B (AKT)/mammalian target of rapamycin (mTOR) signaling pathway [44]. Therefore, an IGF-I serum level within the upper part of the normal range has been associated with an increased risk of cancer development. In tumor cells, including in CRC and liver cancer, fetal isoforms of the IR have been observed to be increased. Leptin, a hormone produced by the adipose tissue stimulates cell growth, migration, and cytokines production by macrophages. Moreover, leptin activates proangiogenic factors, being also involved in tumor development [44]. Some cancer cells, such as those from human CRC, can locally produce IGF-II, triggering tumor proliferation and further metastatic effects [44].

Leptin and adiponectin are involved in cancer cell proliferation, invasion, and metastasis by the activation of the Janus kinase (JAKs)/signal transducer and activator transcription proteins (STATs), mitogen-activated protein kinase (MAPK), PI3K, mTOR, and the AMP-activated protein kinase (5’AMPK) signaling pathways and induce multiple dysregulations, including those of Cyclooxygenase 2 (COX-2) and mRNA expression [45].

The adipose tissue can produce pro-inflammatory cytokines (Interleukins-ILs, IL-8, IL-6, and IL-2), enzymes (lactate dehydrogenase-LDH) and tumor necrosis factor alpha (TNF-α). The lipid peroxidation process leads to 4-hydroxynonenal (4-HNE) formation, an active compound that upregulates prostaglandin E2, which is directly correlated with an increased risk of CRC development. Furthermore, 4-HNE can dysregulate cell proliferation, cell survival, differentiation, autophagy, senescence, apoptosis, and necrosis via MAPK, PI3K/AKT, and protein kinase C signaling pathways [46].

Moreover, the adipose tissue of obese patients present M1 macrophage, which will secrete tumor-promoting molecules, such as TNF-α, IL-1β, IL-6, IL-8, IL-18, IL-32, interferon (IFN)-γ, vascular endothelial growth factor (VEGF), osteopontin (OPN), tenascin C (TNC), and monocyte chemoattractant protein (MCP)-1 [47]. During cancer development, TNF-α is involved in cellular transformation, promotion, survival, proliferation, invasion, angiogenesis, and metastasis [48].

Soltani G et al. conducted a study that included 693 patients who were evaluated for adenoma/adenocarcinoma and underwent colonoscopy. The study concluded that obese and diabetic patients present an increased risk of developing adenoma versus the control group. The research group did not detect any association between obesity, diabetes, and adenocarcinoma [49]. Another important risk factor for CRC may be considered the gut microbiota disruption [50]. Diet can influence the gut microbiota through production of metabolites. Butyric acid, an important source for colonocytes, protects the colonic epithelial cells from tumorigenesis, having anti-inflammatory and antineoplastic properties. Instead, protein fermentation and bile acid deconjugation will damage the colonic cells in proinflammatory and pro-neoplastic ways, leading to increased risk of developing CRC [51]. Moreover, the initial microflora plays a key role in maintaining the survival and health of the host organism, because it can activate antitumor cytokines and reduce the production of oxygen free radicals. In CRC patients, a significant intestinal decrease in intestinal microbiota diversity versus healthy people has been observed. Moreover, intestinal microbiome dysregulation can stimulate intestinal epithelial cells to activate the nuclear factor kappa-light-chain-enhancer of activated B cells (NF-κB) pathway that will trigger an inflammation stage [52]. Dysbiosis or imbalance of gut microbiota may cause chronic inflammation, which is recognized as one of the prime causes of CRC. Therefore, gut microbiota-derived phytometabolites can eliminate gut pathogenic organisms and reduce DNA oxidative damage and pro-inflammatory mediators, regulating normal cell division and apoptosis [53].

Patients diagnosed with long-standing ulcerative colitis and Crohn’s disease have an elevated risk of developing CRC [54]. Furthermore, gut microbiota has effects on the immune cells in the lamina propria, which further influence the inflammation process and subsequently CRC [55]. The fermented fibers produce butyrate, which further induces tumor cell and T-cell apoptosis, which represents the source of colonic inflammation [56]. Saturated fats or the Western diet negatively alter the gut microbiota. Instead, a diet rich in n-3 PUFA has a positive effect on gut microbiome, increasing the production of good probiotics such as *Lactobacillus* and *Bifidobacteria* and reducing *Helicobacter and Fusobacteria nucleatum* [56]. Smoking is another risk factor for CRC, especially in the case of individuals that have smoked for over 30 years [57]. Moreover, bile acid synthesis, such as cholic acid, may be strongly associated with colon cancer development [58].

## 3. CRC Pathogenesis

According to the Cancer Genome Atlas, three molecular types of CRC tumors, hypermutated (13%), ultra-mutated (3%), and with chromosomal instability (CIN) (84%), have been identified [59]. CIN-CRC type is associated with inactivation or loss of *Adenomatous polyposis Coli (APC*) tumor suppressor gene as an early event in neoplasia development. The hypermutated-CRC type is characterized by DNA mismatch repair (MMR) and microsatellite instability (MSI) and is often associated with wild-type *TP53* gene mutation [60]. In total, 70% of CRC adenomas are correlated with early *APC* gene mutation, which usually progress to carcinoma by acquiring *KRAS* as well as *TP53* and *SMAD4* inactivated mutations. Moreover, a small subset of sporadic CRC cases has active *BRAF* mutations [61]. Approximately 15% of CRC have MSI due to either epigenetic silencing of *MLH1* or a germline mutation in one of the mismatch repair genes *MLH1, MSH2, MSH6*, or *PMS2* [62].

More than 80% of sporadic CRC cases manifest CIN and are characterized by chromosome changes such as gains, deletions, and translocations [59]. In sporadic CRC adenomas and adenocarcinomas, *APC* gene mutations are frequently reported as being nonsense or frame shift mutations that encode for truncated APC proteins [63]. CRC adenoma–carcinomas that are observed in most human CRC cases are 84% CIN tumors with DNA somatic alteration and mutations in *APC, TP53, KRAS, SMAD4*, and *PIK3CA* genes [59]. The *KRAS* gene, also known as *Kirsten Rat Sarcoma Viral* oncogene homologue, is located on human chromosome 12, which encodes for the KRAS protein [64]. Wang D and colleagues detected in 6364 CRC tumors that *KRAS* mutation is abundant among Chinese patients [65]. Familial adenomatous polyposis (FAP), which has an increased risk of CRC progression, is mainly caused by *APC* gene mutation [66], which may account for 87% and which are causative point mutations, while 10%–15% of them are intragenic deletions and duplications [67].

Currently, *APC* gene has become one of the most frequent mutations in CRC patients with a family history of polyposis [68]. Ye ZL and co-workers published the results of a study conducted over a period of 20 years (May 1998–December 2018), which included 1190 Chinese CRC patients who had undergone clinical genetic testing. The study reported that 582 CRC patients (48.9%) had gene mutations, among whom 19.7% presented two concurrent mutations and 1.0% with three concurrent mutations. The most common gene mutations were *KRAS* (36,1%) followed by *PIK3CA* (10.2%), *NRAS* (3.9%), *BRAF* (2.9%), *HRAS* (0.9%), and epidermal growth factor receptor *(EGFR*) (0.9%). Regarding the relationship between mutation and prognosis, the study did not find any association between *KRAS/NRAS/PIK3CA/BRAF* mutations and CRC prognosis. Instead, *BRAF* mutation was associated with poor prognosis in CRC patients who received anti-EGFR therapy [69].

The study conducted by Yaeger R and colleagues reported oncogenic alterations in *KRAS, BRAF, PIK3CA, AKT1, RNF43,* and *SMAD4* in 1,134 mCRC patients with right-sided primary tumor compared with left-sided primary tumors [70].

Paerlman R and his research team investigated the rate of gene mutations in 450 patients with early-onset CRC (younger than 50 years). The study reported that 16% of patients have 75 gene mutations. In addition, from 48 patients (10.7%) with MMR-deficient tumors, 40 patients (83.3%) had at least 1 gene mutation, and, from 402 patients (89.3%) with MMR-proficient tumors, 32 patients (8%) with at least 1 gene mutation [71]. Wang Q and co-workers reported that the *PIK3CA* gene mutation was 9.55% in 440 CRC patients. They also showed worse response to first-line chemotherapy, because this mutation causes PI3K/AKT signaling pathway activation, which increases LGR5^+^ CRC stem cells survival and proliferation [72].

In addition, activation of *KRAS* mutations has been reported in various malignancies involved in cell proliferation, anti-apoptosis, and angiogenesis. More than 40% of *KRAS* mutations have been detected in CRC [64]. The *RAS* genes family, which includes *KRAS*, *NRAS*, and *HRAS*, plays crucial roles in EGFR-activated signaling pathways [46]. *APC* gene mutations promote β-catenin dysregulation, which further activates the wingless-type (Wnt) pathway; therefore, the mechanism of polyps’ formation is activated leading to cancer progression [73]. In CRC, *NRAS* mutations are shown in about 3–5% of cases, while *HRAS* mutations are negligible events [74]. 

Recently, in eukaryotic cells, circRNAs have been detected, which are a class of ubiquitous and abundant RNA molecules, characterized by the absence of both 5′caps and 3′tails. CircRNAs play key roles in cancer growth, metastasis, stemness, and resistance to therapy, including CRC [75]. Therefore, during CRC progression, genetic alterations occur in the initiation, transformation, and progression steps of normal colonic stem cells into neoplastic, malignant, and metastatic cells [76].

An increased risk for CRC and polyposis formation is the germ-line mutations in the exonuclease domain of DNA polymerase Pol δ and Pol ε. Moreover, two recurrent pathogenic variants, *POLE* p.L424V and *POLD1* p.S478N, have been identified in CRC family cases [77]. On the other hand, stool DNA testing is more sensitive than the fecal occult blood test. Stool DNA testing is a noninvasive procedure based on colonocytes exfoliation from malignant lesions, which are higher compared with normal tissue [78].

From the molecular point of view, CRC has been classified in 4 consensus molecular subtypes (CMS). CMS1 presents MSI status, *BRAF* mutation, increased immune cell infiltration, and upregulation of checkpoint inhibitors, while CMS2 is characterized by CIN, Wnt/MYC pathway activation, and decreased immune cells infiltration. The CMS3 subtype has *KRAS* mutation, whereas CMS4 has a mesenchymal phenotype with transforming growth factor-β (TGF-β) activation and a high rate of stromal and immune cell infiltration [79]. The TGF-β family of cytokines inhibits normal growth of epithelial cells and may promote tumorigenesis when they lose their sensitivity. After the binding of TGF-β ligands to TGF-β type I and type II receptors (TGFBR1, TGFBR2), TGF-β signaling is activated, which further phosphorylates the receptor-activated SMADs (R-SMADs), SMAD2, and SMAD3, involved in transcriptional regulation. TGF-β pathway members’ mutations are common in multiple human types of malignancies including CRC. Approximately, 10% and 15% of patients with sporadic CRC cases have *SMAD4* and *TGFBR2* mutations, respectively. Moreover, *TGFBR2* mutation is particularly present in MSI tumors [80].

Calcium-activated chloride channels (CLCA) are proteins involved in chloride transport across the plasma. The CLCA family proteins have 3 subtypes (CLCA1, CLCA2, and CLCA4) that have a high degree of homology regarding sequence and functions, but with differences in tissue distributions. CLCA4 expression is downregulated in human cancers including CRC. In CRC and many other cancers, CLCA4 mutation has a decreased prevalence (0.44% of CRC) [81].

The complex cooperation between tumor cells with stroma, immune cells, and endothelial cells will constitute the tumor microenvironment (TME) [82]. TME orchestrates the tumor proliferation, immune evasion, metastasis, and chemoresistance. The stromal cells, extracellular matrix (ECM) components, and exosomes are the main TME components. Endothelial cells, cancer-associated fibroblasts, pericytes, immune cells, lymphocytes, natural killer cells, regulatory T cells (Treg), tumor-associated macrophages (TAMs), myeloid-derived suppressor cell chemokines, matrix metalloproteinases-MMPS, and integrins can be detected in CRC-TME [83]. These cells suffer dynamic changes that will sustain the progression and metastasis of CRC tumors [83]. The myeloid cells sustain the survival and proliferation of neoplastic cells by the inflammatory cytokines (IL-6, IL-1, IL-23, and IL-17A) release or may induce an adaptive anti-tumoral immunity (IL-12, interferon gamma-IFN-γ) [84]. Zhang R and co-workers conducted experimental studies (in vivo and in vitro) and reported that cancer-associated fibroblasts attract monocytes by secreting IL-8 and subsequently promote M2 polarization of macrophages correlated with the suppression of the function of natural killer cells. In addition, IL-6 secretion promotes the adhesion between monocytes in CRC cells [85]. 

Therefore, TME has pro-tumorigenic effects through cytokines and growth factors (GFs) that will support cancer cell proliferation, survival, motility, and invasion [82]. The presence of inflammatory cells and inflammatory mediators such as chemokines and cytokines will facilitate CRC progression. Moreover, a single cytokine can activate signaling pathways, leading to tumor progression and development [86]. IL-6 level is increased in CRC patients’ serum versus those of healthy subjects. Moreover, studies performed in vitro and in vivo revealed that IL-6 stimulates the invasiveness of human CRC cells, promoting colonic tumor growth [87]. Chemokines are small proteins that can bind to G-protein-coupled receptors that are involved in tumorigenesis, metastasis, and angiogenesis. However, there are chemokines with a positive impact. For example, CC ligand 19 (CCL19), also named as macrophage inflammatory protein 3-beta (MIP-3b), inhibits tumorigenesis, metastasis, and angiogenesis, and is associated with a good prognosis of CRC patients. Studies in vivo and in vitro performed by Xu Z et al. revealed that, in CRC cases, CCL19 may block angiogenesis by inhibiting tyrosine-protein kinase Met (Met)/extracellular signal regulated kinase (ERK)/Elk-1/hypoxia-inductible factor-1 alpha (HIF-1α)/VEGF-A pathway in a CCR7-dependent pattern [88]. 

De la Fuente López M and his research team have evaluated the levels of chemokines (CCL2, CCL3, CCL4, CCL5, and CX3CL1), TNF-α, and VEGF, in both plasma and tissue lysates of 48 CRC Chilean patients. Chemokines, TNF-α, and VEGF levels from tissue lysate of CRC patients statistically increased compared with healthy tissue. The plasma levels of CCL2, CCL3, CCL4, TNF- α, and VEGF were detected in 32 patients with CRC and 15 heathy subjects. From all chemokines measured, only CCL3 had a statistically higher level in CRC patients’ plasma. The research team observed positive correlations between the plasmatic level of CCL4 with TNF -α and VEGF, correlations that reflect poor prognosis of CRC patients. Therefore, plasmatic levels of chemokines together with TNF-α and VEGF can be used as biomarkers for CRC prognosis [89].

Macrophages support neoplastic transformation and malignant progression by ROS release, which will be conducive to DNA damage and mutation in neighboring epithelial cells. Moreover, via NF-kB pathway, commensal bacteria, and microbial products induce the secretion of inflammatory cytokines, including IL-1β, IL-6, and IL-23, which further promote the proliferation and survival of neoplastic cells and pro-tumorigenic Th-17 T cells differentiation [90]. In CRC microenvironment, TAMs shift from M1 to M2 macrophages, which are induced by Th2 cytokines [91]. M1 macrophages possess anti-tumor properties, while M2 macrophages lead to immunosuppression and tumorigenesis [91]. CRC patients with M1 macrophages infiltration at the tumor site have been observed to be correlated with a better prognosis. Unfortunately, most CRC TAMs display the M2 phenotype [92].

The epithelial–mesenchymal transition (EMT) plays an important role in the metastasis process, being involved in the interaction between the tumor cells and TME. Moreover, EMT-programmed tumor cells release inflammatory mediators that change the cellular and noncellular components of TME [93]. Moreover, cytokines released from infiltrated inflammatory cells contribute to tumor initiation by ROS and reactive nitrogen species (RNS) increased levels production, because they change the epigenetic of tumor suppressor genes. On the other hand, cytokines and chemokines sustain tumor growth in the later stage of tumorigenesis by promoting angiogenesis and suppressing the anti-tumor immune response [94].

During EMT transition, the epithelial cells lose the epithelial phenotype and acquire the mesenchymal phenotype [95]. Tumor cells, including CRC, undergo metabolic reprogramming, including glycolysis, mitochondrial energy production, lactate, and fatty acid metabolism important for the maintenance of malignant features, which will lead to a rapid proliferation rate [96]. microRNAs (miRNAs) are important regulators of CRC metabolic reprogramming, which sustain the metabolic processes after interactions with enzymes, transporters, suppressors, and oncogenes. Moreover, due to its localization in CRC epithelial cells, MiR-181a detection can be a valorous prognostic biomarker for mCRC patients, which is correlated with distant metastasis and poor overall survival [96].

Under various stimuli, EGFR signaling regulates macrophage activation. EGFR phosphorylation occurs in macrophages and will have major effects on the expression of both M1 and M2 macrophages [97]. EGFR signaling has been mostly studied within the context of epithelial cell function and has been correlated with CRC initiation and progression [97]. Besides EGFR, VEGF receptor (VEGFR) is mostly expressed in endothelial cells including CRC [98].

**Figure 1:**
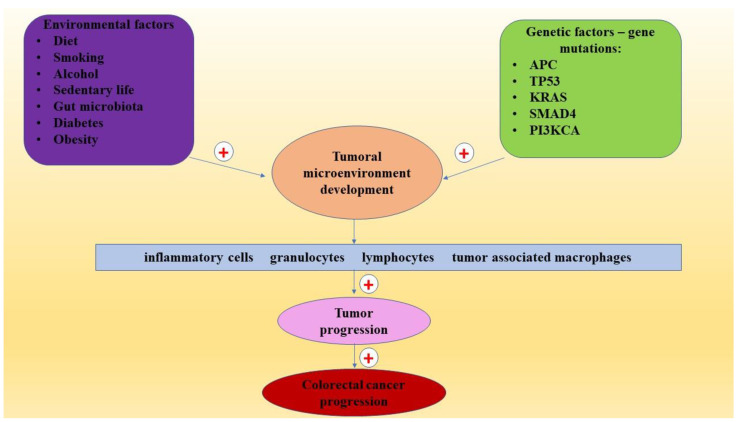
The environmental and genetic factors involved in colorectal cancer progression.

In addition, obesity characterized by chronic inflammation contributes to CRC progression by several mechanisms, including insulin, IGF, leptin, adiponectin, microbiome, and cytokines [99]. The most important environmental and genetic factors involved in CRC pathogenesis are presented in Figure 1.

### 3.1. CRC and Insulin-Like Growth Factor Family (IGF)

The IGF family of proteins have three ligands, IGF1, IGF2, and insulin, which will bind to the following surface transmembrane receptors: IGF1R, IGF2R, and insulin receptor (IR) [100]. IGF-1 receptor (IGF-1R) is a transmembrane glycoprotein that acts as a tyrosine kinase receptor and presents two extracellular units and two cytoplasmic subunits [101]. Moreover, it is involved in many human cancers, favoring cell growth, proliferation, differentiation, apoptosis, and angiogenesis [101]. IGF-1R overexpression has been detected in CRC, pancreatic, gastric, and esophageal cancer [102]. In addition, IGF-1 and IGFBP-3 favor angiogenesis by increasing the VEGF gene transcription. An elevated serum ratio for IGF-1/insulin-like growth factor binding protein-3 (IGFBP-3) was associated with increased risk of CRC [103]. Both IGF-1 and STAT3 can induce CRC development and progression via cell-autonomous and microenvironmental effects [82]. During cancer progression and metastasis, insulin and IGF-1 have a functional role, especially in patients with hyperinsulinemia. Furthermore, insulin is able to induce mRNA expression of the matrix metalloproteinase-2 (MMP-2) by activating the signaling pathways insulin receptor substrate-1 (IRS1)/PI3K/ AKT and MAPK signaling in HCT-116 human colorectal cells [104]. Both MMP-2 and MMP-9 are involved in the regulation of the activity of cell receptors and growth factors. Moreover, MMP-2 is overexpressed in tumor tissues, including CRC [105]. The expression of IGF-1R is found in mild, longstanding inflamed colon, which will further lead to elevated levels of both mARN and protein. In these inflammatory conditions, epithelial cells may suffer pathological changes [106]. Moreover, in murine acute colitis, IGF-1-primed macrophages will suppress intestinal immune inflammation by producing IL-10 [106]. In inflammatory conditions, immune and epithelial cells release ROS and nitrogen species (RNS), which will induce DNA lesions [59]. Currently, IGF-1R has been recognized as a major determinant of cancers, while its biological roles and exact tumorigenesis mechanisms remain elusive [64]. IGF-1R plays crucial roles in mitochondrial respiratory chain regulation, which is a key element between colitis and CRC development [107]. IGF-1R, together with mesenchymal-epithelial transition (MET), is frequently overexpressed by various tumor types, including CRC [108]. Additionally, IRS-1 may present a certain association with colon cancer incidence [108]. Jiang B et al. reported that serum levels of leptin, insulin, IGF-1, and IGF-1/IGFBP3 in CRC patients were significantly elevated compared with healthy ones, while the IGFBP-3 level decreased compared with controls. These aspects suggest that serum detection of IGF-1 may be an early warning indicator [109]. To test the implication of hyperinsulinemia in CRC progression, various epidemiologic observations and experimental studies were performed [109,110]. Dietary-induced hyperinsulinemia and hypertriglyceridemia may affect the colon by producing aberrant crypt foci, a putative precursor of colon cancer [110]. Moreover, it has been observed that insulin influences the growth of the colon epithelial and carcinoma cells in vitro [110]. Hu J and his research team measured the expressions of IGF-1, ERK, GLUT4, and IRS-1 in CRC patients with metabolic patients compared with healthy controls [111]. The study concluded that the expression levels of IGF-1 and ERK were elevated in patients with metabolic syndrome with/without CRC versus the healthy controls [111]. The expression of GLUT4 was decreased in CRC patients with metabolic syndrome, compared with patients without metabolic syndrome and controls [111]. Moreover, the study observed that expression levels of ERK, IGF-1, and GLUT4 were correlated with CRC clinical characteristics, such as tumor size, distant metastasis, and advanced stages (III/IV) [111]. Peters G and co-workers detected the expression of IGF-1, IGF-2, and IGF-1R in CRC patients [112]. The expression of IGF-1 was observed in 7.5%, IGF-2 in 12.6%, while IGF-1R in 99.6% of the cases [112]. Moreover, the study detected few associations between IGF-1 and Ki-67, IGF-2, and tumor stage. In addition, IGF-2 was positively correlated with worse clinical outcomes [112]. Alagaratnam S et al. detected IGF-1Ec, an isoform of IGF-1, in 16 patients with CRC and 11 patients with colonic polyp. IGF-1EC has been identified to be overexpressed in cancers, such as prostate and neuroendocrine tumors [113]. The study revealed a significantly increased expression of IGF-1Ec in CRC patients (*p* < 0.001) and colorectal polyps (*p* < 0.05) compared with normal colonic tissues [113]. Furthermore, it has been postulated that markers of hyperinsulinemia such as IGF-1 and C-peptide may be correlated with an increased risk of CRC [114]. In addition, phosphorylated nuclear IGF-1R (nIGF-1R) is expressed in approximately 20% of mCRC and 50% of patients harboring mutations within the *BRAF* gene [115]. The inhibition of IGF-1/IGF-1R signaling will inactivate downstream AKT/mTOR signaling pathway [115].

### 3.2. CRC and Epidermal Growth Factor (EGF)

Dysregulation of the EGF receptor (EGFR) signaling pathway is frequently met in human cancers, including CRC [116]. The EGFR (ERB-1 or HER-1) is a member of the human EGFR (HER)-erbB family of tyrosine kinase receptors (RTKs), which includes three other members, such as HER2/C-neu (ErbB2), HER3 (ErbB3), and HER4 (ErbB4) [117]. EGFR is a glycoprotein that belongs to the ErbB family member of RTK, which presents an extracellular ligand-binding domain and an intracellular tyrosine kinase domain [118]. In the absence of specific ligands, such as EGF, TGF-α, epiregulin (EREG), betacellulin, heparin-binding EGF-like growth factor (HB-EGF), amphiregulin (AREG), epigen, heregulin, and neuregulins 1–4, EGFR is in a state of inhibition [118]. After the binding of one of the mentioned ligands to the extracellular domain, homo- or hetero-dimerization takes place, triggering the phosphorylation of the tyrosine kinase domain and activation of the RAS-RAF-MAPK signaling pathway, promoting tumor growth and progression [118]. EGFR can be found on the cell membrane surface, and its expression is elevated in cancer, moderate in adenoma, and very decreased in normal epithelia [119]. EGFR is an excellent candidate for targeted cancer therapy, being over-expressed in many types of cancers, including CRC [120]. Moreover, after binding to its receptor EGFR, EGF will activate the PI3K/AKT/mTOR signaling pathway, which is critical to cell survival, motility, and invasion [118,121]. Moreover, in CRC, EGFR mutation is rare [121]. The survival of patients with mCRC has been significantly improved with the introduction of the monoclonal antibodies that have as target EGFR [122]. The human epidermal growth factor receptor (HER-2) protein is involved in cancer cell proliferation, differentiation, and apoptosis [123,124]. HER-2 is a transmembrane tyrosine growth factor receptor that is considered to be a relevant therapeutic target in several human cancers, including CRC [123,124]. Moreover, HER-2 can be found on normal and malignant epithelial cells [123,124]. Lawan AI and co-workers explored the expression of EGFR in 54 patients with CRC carcinoma and reported that EGFR was expressed in 85.2% of the cancer cases [125]. Moreover, the study observed an association between EGFR status and depth of tumor invasion and tumor size. EGFR presence is correlated with a poor survival rate [125]. EGFR contributes to malignant behaviors of colon cancer cells in five ways—transformation of non-tumorigenic cells into tumorigenic cells, mitogenesis of polarizing colon cancer cells, cancer cells proliferation, cellular metastasis, and autophagy [126]. In addition, the tumorigenic effect of EGFR is attenuated in the presence of TGF-β signaling. Therefore, TGF-β may stimulate EGFR to create a beneficial microenvironment for metastasis [126]. Nemanqani DM and colleagues explored the expression of EGFR in 35 CRC specimens and observed its presence in 74% of the studied specimens [127]. The study also observed a higher EGFR expression mostly in grade II (85%) and stage T3 of tumors (69%) [127]. Thus, CRC EGFR over-expression could be a biomarker for an unfavorable prognosis [128].

### 3.3. Colorectal Cancer and Vascular Endothelial Growth Factor (VEGF)

Angiogenesis, the process of formation of new blood vessels, is fundamental for the growth of all tumor cells, including CRC [129]. VEGF is a member of the platelet-derived growth factor family that includes related glycoproteins, such as VEGF-A, VEGF-B, VEGF-C, and VEGF-D [130]. VEGF is one of the most important and specific factors that stimulate angiogenesis in both situations, physiological and pathological [131]. In addition, VEGF is excessively synthetized in epithelial, mesenchymal, and particularly in tumor cells. Additionally, elevated serum levels of IL-6, TNF-α, and VEGF are strongly associated with CRC and with the clinical stage of this disease [132]. VEGF has two receptors, VEGFR1 and VEGFR2, that act through tyrosine receptor kinases, which are implicated in angiogenesis, while VEGFR3 is involved in lymphangiogenesis [133]. However, VEGFRs are not only expressed in vascular endothelial cells, but also by the macrophages and monocytes [133].

VEGF regulates angiogenesis and vascular function. Thus, VEGF can promote angiogenesis in various pathologic conditions, including cancer, mediating endothelial cell proliferation and survival [134]. Mohamed SY et al. evaluated the expression of VEGF in 50 patients diagnosed with CRC [135]. VEGF was expressed in 70% of the cases, and presented a significant correlation with tumor size, grade, and advanced tumor stage [135]. Unfortunately, VEGF-A is correlated in CRC patients with poor clinical outcome, mainly in stages II and III [136]. Moreover, VEGF-A may be a prognostic factor in mCRC patients [137]. Jannuzzi AT et al. evaluated the VEGF single-nucleotide polymorphisms (VEGF −2578A > C, +936C > T, and −460C > T) in patients diagnosed with CRC [138]. The study illustrated that VEGF-2578A > C was significantly associated with CRC risk, while +936C > T and −460C > T genotypes did not present significant differences between CRC patients and controls [138]. Therefore, VEGF polymorphisms might play a role in CRC development [138]. VEGFA knockdown could inhibit CRC cell growth [139]. Moreover, EGFR and VEGF can be detected in CRC patients using fluorescence-Raman endoscopy [140]. In addition, VEGF-A expression in CRC tissue is associated with worse survival rate in male compared with females [141].

## 4. PI3K/AKT/mTOR and MAPK Signaling Pathways in Colorectal Cancer

PI3Ks are intracellular lipid kinases that are implicated in regulation of cellular proliferation, differentiation, and survival [142,143]. PI3K/AKT/mTOR signaling pathway overexpression has been reported in various cancers types, including CRC [142,143]. It is well known that PI3Ks are kinases promoting cellular proliferation [144]. Mutations that occur in *PIK3CA* gene encoding p110α catalytic subunit of PI3K have been detected in different human solid tumors, including CRC [144]. PI3K/AKT/mTOR signaling pathway plays a crucial role in cancer development including proliferation, metastasis, survival, and angiogenesis [144]. Moreover, AKT and mTOR are both downstream targets of VEGF-A [144]. Beside PI3K/AKT/mTOR signaling pathway, all the three major subfamilies of MAPK—ERK, the c-Jun N-terminal kinase or stress-activated protein kinases (JNK or SAPK), and MAPK14—are involved in CRC pathogenesis [144]. The ERK/MAPK plays a key role in cell proliferation. Moreover, the MAPK pathways are situated downstream of many GFs receptors, including EGF [144]. Therefore, the MAPK pathways are activated by various stimuli, such as peptide growth factors, cytokines, hormones, oxidative stress (OS) and endoplasmic reticulum stress, regulating cells’ proliferation, differentiation, survival, and death [145]. The ERK signaling pathway plays a crucial role in tumorigenesis, including cancer cell proliferation, migration, and invasion, including in CRC [145]. In CRC tumors, the *PIK3CA* gene mutation has been identified in 10–20% of cases [146]. EGFR is a valuable therapeutic target in mCRC [147]. EGFR influences the tumorigenic cells’ proliferation by activation of ERK1/ERK2, which is stimulated by Src, which further mediates a cross talk between EGFR and aryl hydrocarbons [3]. Therefore, MAPK and PI3K/AKT signaling pathways are responsible for cancer cell survival and invasion [148]. The Raf/mitogen-activated protein kinase (MEK)/ERK signaling pathways transmit signals from GFs receptors and further regulate gene expression and may prevent apoptosis [149]. After VEGF binds to VEGFR-2, phosphorylation at specific tyrosine residue occurs, and further activation of ERK1/ERK2 rapidly accelerates fibrosarcoma Raf/MEK1-MAPK, triggering increased cell proliferation [150]. mTOR pathway inhibition may induce suppression of invasion and migration of tumoral cells [151]. EGFR activates PI3K, which further catalyzes the phosphorylation of PIP_2_ (phosphatidylinositol 4,5-bisphosphate) to PIP3 (phosphatidylinositol 3,4,5-triphosphate), an important second messenger involved in AKT recruitment, which activates mTOR, involved in the activation of growth, proliferation, and survival signaling responses [152]. The negative regulator of PI3K/AKT signaling cascade, Phosphatase and Tensin Homolog (PTEN), dephosphorylates PIP3 to PIP2 and is over-expressed in human colon cancer in around 60–70% patients [153,154]. In addition, increased levels of EGF trigger synthesis of hydrogen peroxide (H_2_O_2_), which stimulates Ribosomal protein S6 kinase beta-1 (S6K1) or p70S6K1 via the PI3K/AKT/mTOR signaling pathway, leading further to VEGF activation [155]. 

## 5. PI3K/AKT/mTOR and MAPK Signaling Pathways Inhibitors

**Figure 2:**
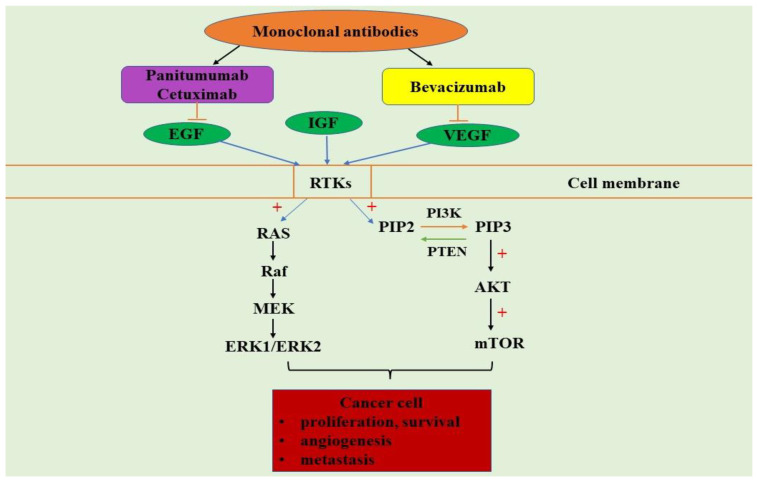
Growth factors, growth factor inhibitors, and PI3K/AKT/mTOR-MAPK signaling pathways in colorectal cancer development.

Surgery, radiotherapy, and chemotherapy are the primary methods for treating CRC [154]. These medical techniques are accompanied by side effects, including reduced gastrointestinal function, reduced immunity, and increased pain after radio- or chemotherapy [154]. The most commonly used target drugs for CRC therapy are those that target EGFR and VEGFR [154]. Important drugs for CRC treatment include monoclonal antibodies and tyrosine kinase inhibitors that have been developed to inhibit EGFR, VEGF, and VEGFR [156]. Being involved in CRC progression, EGFR is an attractive target for therapy acting on monoclonal antibodies and the tyrosine kinase inhibitors [157]. The monoclonal antibodies used to target EGFR have been applied in mCRC treatment, with good results for patients [158]. In patients with mCRC, the anti-EGFR antibodies, cetuximab (an IgG1 recombinant human/mouse chimeric anti-EGFR mAb) and panitumumab (an IgG2κ recombinant, only human anti-EGFR mAb), have been used in several phase III clinical trials [159]. These antibodies present efficacy in terms of progression-free survival (PFS) and overall survival (OR) and are able to prolong patients’ survival when are used as monotherapy or in combination with other drugs [159]. Cetuximab and panitumumab are target drugs against EGFR, while bevacizumab, ramucirumab, zivaflibercept, and regorafenib act against VEGF [160]. Among all, bevacizumab is the only VEGF-targeted agent approved by the US Food and Drug Administration (FDA) for mCRC patients [160] (Figure 2). In the case of CRC patients with extended *RAS* wild-type profiles, and with left-sided tumors, the EGFR antibodies therapy should be restricted [161]. The molecular alterations of the oncogenes such as *RAS*, *BRAF*, *PI3KCA*, and PTEN in the downstream pathway of EGFR, which activates MAPK/ERK signaling pathway, represent the novel mechanisms of resistance to anti-EGFR therapies [162]. Studies reported that among patients with CRC tumors carrying wild-type *KRAS*, *EGFR* gene copy number, mutations of *BRAF*, *PIK3CA*, or loss of PTEN expression develop resistance to anti-EGFR therapy [117]. The meta-analysis conducted by Therkildsen C and his research team demonstrated that mutations in *KRAS*, *NRAS*, *BRAF*, *PIK3CA*, and loss of PTEN will predict resistance to anti-EGFR therapies in the case of mCRC patients [163]. Canavese M et al. reported that EGFR therapy with monoclonal antibodies (cetuximab and panitumumab) improves outcomes in mCRC patients with wild-type *RAS* oncogene [164]. The treatment with the anti-EGFR moAb cetuximab activates the RAS-RAF-MEK-MAPK pathway, which is the main EGFR downstream effector [164]. Napolitano S et al. evaluated the cetuximab resistance in various human CRC models in combination with MEK inhibitors (MEKi) [165]. The in vivo and in vitro results performed on a CRC model demonstrated that the combined treatment between cetuximab and MEKi has synergic anti-proliferative and pro-apoptotic properties, combined with MAPK and PI3K/AKT/mTOR inhibition [165]. The anti-VEGF-A monoclonal antibody (Mab), bevacizumab, or Avastin was approved by the FDA for the treatment of mCRC [166]. Therefore, bevacizumab is used for solid tumor types and currently is the most widely used cancer therapeutic drug. Studies have shown that bevacizumab has a significant survival rate in patients with previously untreated mCRC when it is combined with fluoropyrimidine [166]. Furthermore, bevacizumab is the first therapy line against mCRC, demonstrating the fact that VEGF is a key mediator of tumor angiogenesis, and blocking angiogenesis is an important strategy to treat human cancer [167]. Currently, in clinical practice, EGFR is targeted by cetuximab, and VEGF by bevacizumab [140]. The detection of plasma or serum concentration of VEGF-A have been analyzed in relation to drug efficacy. The results were contradictory—after the bevacizumab treatment, the levels of serum VEGF-A may be decreased. But an elevated serum level of VEGF-A after an initial decrease has been associated with a poor response and a reactive resistance to chemotherapy with bevacizumab [168]. Bevacizumab-VEGF inhibitor, in combination with other anti-angiogenic agents (murine inhibitor) and ONC201 in both CRC xenograft and patient-derived xenograft (PDX) models, may lead to significant tumor regression or even complete tumor ablation [169]. Fruquintinib may be a promising oral drug in the CRC fight, being an active inhibitor of VEGFR-1, -2, -3 tyrosine kinases, inhibiting VEGFR-2 phosphorylation, endothelial cell proliferation, and tubule formation. Presently, it is used in China for mCRC treatment in patients that have failed at least two prior systemic antineoplastic therapies [170]. The resistance that appears in VEGFR inhibitors seems to be attributed to receptor mutations that appear in PIK3CA/AKT, ERK, HER-2, or even EGFR [171]. The effectiveness of two monoclonal antibodies, cetuximab and panitumumab, increases in combination with fluorouracil (5-FU) plus irinotecan (FOLFIRI) and 5-FU plus oxaliplatin (FOLFOX) by acting on EGFR, leading to RAS-RAF-MEK-ERK signaling pathway inhibition in mCRC patients [172]. 5-FU has been used in the medical practice for the management of CRC for decades and is now utilized in combination with other chemotherapeutic agents that may activate MAPK [173]. CRC patients may develop resistance to chemotherapeutic drugs, including cisplatin, irinotecan, and 5-FU, due to MAPK, p38α MAPK being a mediator of resistance [174]. p38 MAPKs have a dual role—they may mediate cell survival or promote cell death through different mechanisms [174]. Based on these aspects, the CRC growth in vitro and in preclinical models is significantly reduced by the combination of the following drugs, such as p38α inhibitors (SB202190, SB203580, and BIRB796), autophagy inhibitors (3MA and bafilomycin), MEK inhibitors (PD98059, UO126, and CI-1040), *HER2* inhibitors (lapatinib), multi-kinase inhibitors (sorafenib), or chemotherapeutic agents (5-FU, irinotecan, and cisplatin), which promote a higher rate of apoptosis versus the single treatment [174]. Cheng H and co-workers tested on CRC cell line the inhibitory effect of Naringin. The research team illustrated that Naringin stops the proliferation of CRC cells, promoting apoptosis by inhibiting the PI3K/AKT/mTOR signaling pathway in a dose-dependent manner [154]. Wang J and his research team tested on seven different colorectal cell lines the effect of W922, a novel PI3K/AKT/mTOR pathway inhibitor, as an efficient anti-tumoral. Between all cell lines used, the HCT116 line was the most sensitive to W922 treatment [175]. W922 was able to inhibit HCT116 cell viability and cell proliferation in vitro, in a concentration and time-dependent manner [143]. Under W922 treatment, the suppression of tumor growth was observed, as well as dephosphorylation of PI3K/AKT/mTOR proteins and mTOR inhibition [175]. Moreover, co-treatment of W922 and chloroquine leads to cells apoptosis, thus providing a promising therapeutic strategy for patients diagnosed with CRC [175]. Kallikrein-related peptidase 10 (KLK10) was identified in 1996 as normal epithelial cell-specific 1, involved in cancer development by regulation of cell growth, invasion, and apoptosis [176]. Moreover, using CRC cell lines, a negative correlation has been detected between KLK10 high expression and OR rate. Therefore, knockdown of KLK10 dramatically suppresses cell viability and induces apoptosis in CRC cell lines [176]. KLK10 acts by blocking the PI3K/AKT/mTOR signaling pathway, inhibiting cell growth and glucose metabolism [176]. Helmy MW et al. explored the effects of diosmin (DIO, a natural NF-κB inhibitor) and BEZ-235 (dactolisib, dual PI3K-mTOR inhibitor) in HCT-116 CRC cells [177]. The research team reported that co-administration of both drugs in two combinations inhibited the PI3K/AKT/mTOR/NF-κB signaling cascades, leading to apoptosis and cell proliferation inhibition, and altered the angiogenesis process [177]. Future preclinical and clinical studies must be carried out [162]. The study conducted by Li S et al. evaluated the effect of the extract *Selaginella doederleinii Hieron* ethyl acetate (SDEA) in vitro and in vivo [178]. Using HT29 and HCT116 cell lines, the anti-tumoral effect of SDEA was manifested by cell morphological changes, cell cycle arrest, autophagy, and apoptosis [178]. Moreover, the SDEA extract may induce the loss of the mitochondrial membrane potential, increases the autophagic flux, and will inhibit the PI3K/AKT/mTOR signaling pathways [178]. Therefore, in xenograft tumors, SDEA inhibits the growth in a dose-dependent manner [178]. The experimental studies conducted by Han YH and co-workers, performed on cell lines, explored the inhibitory effect of betulin in mCRC [179]. Studies performed in vitro illustrated that betulin can induce apoptosis, autophagy, and cell cycle arrest by PI3K/AKT/mTOR and MAPK signaling pathways inactivation [179]. In addition, oral administration of betulin significantly inhibits CT26 cell lung metastasis [179]. Li N and his research team evaluated in vitro if nobiletin may enhance the inhibitory effect of oxaliplatin on CRC cell lines [180]. The study reported that nobiletin increases CRC sensibility to oxaliplatin to induce CRC cells’ apoptosis, as evidenced by the increased expression of pro-apoptotic proteins and the downregulation of anti-apoptotic protein Bcl-2 [180]. Moreover, this combination will downregulate the PI3K/AKT/mTOR pathway [180].

Studies performed in vivo observed that MEK and RAF inhibitors suppress colorectal tumor growth, but these cells develop resistance to these inhibitors by activating the PI3K/AKT/mTOR or JAK/STAT signaling pathways that mediate resistance. Understanding the mechanisms of CRC drug resistance will enhance the patients’ survival rate [181]. The FRESCO Randomized Clinical Trial conducted by Li J et al. explored the efficacy and safety of oral fruquintinib, a VEGFR inhibitor, as third-line or later therapy in 519 patients (aged 18 to 75 years) diagnosed with mCRC. The study observed a median OR significantly improved by fruquintinib versus placebo (9.3 months compared with 6.6 months). Moreover, the median PFS was also significantly increased with fruquintinib (3.7 months versus 1.8 months) [182]. PI3K or AKT inhibitors may be used in CRC clinical trial with promising results, but drug resistance frequently appears, driven by β-catenin, which blocks FOXO 3A to induce apoptosis. Therefore, using Wnt/β -catenin signaling pathway inhibitors will reduce PI3K or AKT drug resistance in CRC patients [183]. Arques and his research team explored in clinical trials, which included CRC patients, if Wnt mediates resistance in patients treated with PI3K or AKT different inhibitors. The study used NVP-TNKS656—a Wnt/tankyrase inhibitor, to overcome PI3K or AKT resistance [183]. The study reported good results because Wnt/tankyrase inhibitor promotes apoptosis in PI3K or AKT inhibitor-resistant cells. For CRC patients, PI3K/AKT/mTOR and Wnt/β-catenin inhibitors represent an excellent strategy [183].

Everolimus—an mTOR inhibitor was administered in 12 patients with primary resectable rectal cancer 14 days prior to the start of chemoradiotherapy and continued throughout the four-week course with 5-FU and radiotherapy. The study detected no increase in toxicity at any of the doses with 5-FU and radiotherapy. Moreover, no significant increase in complete pathological response (pCR) was observed and the everolimus maximum tolerated dose was 10 mg. The study concluded that the combination of chemoradiotherapy and everolimus has feasible results over long time. Another mTOR inhibitor, rapamycin, was used in Phase I (13 patients) and II clinical trial (31 patients) with primary resectable rectal cancer, where patients received rapamycin one week before and during radiotherapy. The study illustrated a higher rate of post-operative complications in phase I. Regarding the patients included in phase II, it was observed that rapamycin was feasible correlated with a significant reduction of tumor metabolic activity [184]. Ganesan P and co-workers evaluated, in early-phase clinical trials, 191 CRC patients with diverse mutations, especially *KRAS* and the PI3K/AKT/mTOR inhibitors. Depending on the mutation, the patients received different drug inhibitors, such as for mTOR, PI3K, and AKT. The study concluded that the median PFS for patients with *PIK3CA* mutations and PI3K/AKT/mTOR inhibitors was 1.9 months, while there was no difference in median PFS in patients with *KRAS* mutations compared with patients with wild type *KRAS* [185]. Garrido-Laguna I and colleagues explored, in early-phase trials, the impact of PI3K/AKT/mTOR inhibitors on 238 patients with mCRC, with 51% *KRAS* mutations and 15% *PIK3CA* mutations. The treatment with different PI3K/AKT/mTOR inhibitors presented a limited activity in these patients, because of the MAPK activating mutations [186]. Kyriakopoulos CE et al. conducted a phrase I trial that evaluated the effect of tivantinib and temsirolimus in patients with advanced solid tumors including CRC. The doses administered in this study were overall well tolerated and demonstrated that this combination has an enhanced anti-tumoral activity [187]. In patients diagnosed with mCRC, everolimus—an oral mTOR inhibitor—presents efficacy. Ng K and his research team evaluated, in a sequential phase II study, the effect of everolimus in 100 patients with mCRC, which were refractory to bevacizumab-, fluoropyrimidine-, oxaliplatin-, and irinotecan. The patients received a high weekly dose of everolimus, while the daily dose has a lower concentration. Median PFS and OS were 1.8 and 4.9 months, and 1.8 and 5.9 months, respectively, for the weekly and daily administrated doses. Among the patients who received a daily dose of everolimus, those with *KRAS* mutations had a significantly shorter median OR versus those with wild-type *KRAS* mutations. The daily or weekly dose of everolimus was well tolerated but did not confer a significant efficacy in mCRC [188]. A phase I study included 27 CRC patients with *KRAS* mutations who received the pan-HER inhibitor dacomitinib in combination with MEK1/2 inhibitor PD-0325901. The patients received various drug doses by oral administration in cycles of 28 days, but the results revealed that the mentioned combination was not tolerated by most of the patients. These results may be explained by the activation of PI3K/AKT and MAPK signaling pathways, by the *KRAS* and *PIK3CA* mutations [189]. A phase I trial was initiated with the pan-HER inhibitor afatinib plus the MEK inhibitor selumetinib in 19 CRC patients with *KRAS* and *PIK3CA* wild-type mutations. In peripheral blood mononuclear cells, inhibition of phosphorylated ERK needs a specified concentration for both selumetinib and afatinib. Although the study reported limited clinical efficacy for the two drugs, several side effects have been reported after oral administration [190]. Folprecht G et al. conducted a clinical study that included 47 patients with mCRC who received EKB-569, an EGFR tyrosine kinase inhibitor, in combination with irinotecan, 5-FU, and leucovorin (FOLFIRI). At the recommended dose of EKB-569 (EKB-569/full dose FOLFIRI), the complete inhibition of phosphorylated EGFR occurs. Instead, FOLFIRI alone did not affect EGFR phosphorylation, but may inhibit epidermal proliferation and MAPK [191]. Tabernero J and his research team evaluated the cetuximab efficacy in tissue samples collected during a phase I as first-line therapy in 62 patients with mCRC. The patients received cetuximab monotherapy for 6 weeks, followed by the administration of cetuximab in combination with 5-FU, leucovorin, and irinotecan until disease progression. In 35 mCRC patients, cetuximab treatment was correlated with substantial downregulation of EGFR, MAPK, and STAT3 phosphorylation. The study reported that PFS was longer for patients with KRAS wild-type compared with *KRAS* mutant tumors [192].

Currently, 3 clinical trials are in progress. A phase II, comparative trial, AtezoTRIBE, includes unresectable and previously untreated mCRC patients that have received FOLFOXIRI treatment (fluorouracil, leucovorin, oxaliplatin, and irinotecan) plus bevacizumab up to 8 cycles (the standard treatment) or a combination with atezolizumab (the experimental treatment), followed by the treatment with 5-FU/leucovorin plus bevacizumab with or without atezolizumab according to disease progression. Until now, a few patients reported severe adverse reactions [193]. The second one, conducted by Damato A and his research team, is a prospective, open-label, multicentric phase II trial, which includes patients with mCRC and RAS/BRAF mutations who received, in the first line of treatment, nivolumab in combination with FOLFOXIRI/bevacizumab every 2 weeks for 8 cycles. After that period, the patients intravenously received bevacizumab plus nivolumab for another 2 weeks in a dose that depends on the patient’s weight. The main aim of the study is to enhance the overall response rate from 66 to 80% [194]. The third, conducted by Meric-Bernstam F et al., is a phase 2a, multiple basket study, called MyPathway, which included patients with HER2-amplified mCRC. Initially, the patients intravenously received an increased dose of pertuzumab, and, every 3 weeks, the dose was reduced by half. For trastuzumab, the loading dose was in an increased concentration, followed by every 3 weeks by a reduced dose administrated intravenously. Although some patients reported several adverse reactions, the preliminary results reported that the dual therapy is well tolerated and could represent a favorable therapy for HER2-amplified mCRC patients [195].

Overall, experimental studies and clinical evidence revealed that polyphenols have an important role in CRC chemoprevention and exhibit cytotoxic effects on CRC cells [196].

## 6. Conclusions

Unfortunately, CRC has an increasing incidence among the young population, and adopting a healthy diet correlated with regular medical analysis may decrease the incidence of this malignancy. The CRC pathogenesis is very complex and assumes the presence of many genetic mutations that will be involved in cancer progression. Moreover, CRC progression is influenced by the presence of inflammatory cells and their inflammatory mediators, such as cytokines that can activate signaling pathways, leading to tumoral development. IGF-1R, EGFR, and VEGF can bind to RTKs, which will activate RAS-RAF-MAPK and PI3K/AKT/mTOR signaling pathways, promoting tumor growth, progression, cell survival, motility, and invasion.

Therefore, important drugs that have already been used in CRC clinical trials are EGFR and VEGFR monoclonal antibodies. The anti-EGFR antibodies, cetuximab and panitumumab, are used in phase III trials especially for mCRC patients with good results regarding PFS and OR. FDA approved bevacizumab—a VEGFR inhibitor for mCRC patients that can inhibit angiogenesis, as a key step in cancer therapy. Gene mutations, including *RAS, BRAF, PI3KCA, PTEN*, and *HER-2* activate MAPK or PI3K/AKT/mTOR leading to resistance to EGFR or VEGF therapy. Thus, a combination of EGFR/VEGF with RAS-RAF-MEK-MAPK and PI3K/AKT/mTOR inhibitors have anti-proliferative and anti-apoptotic properties. Currently, the experimental in vitro studies focus on blocking the PI3K/AKT/mTOR/NF-kB and MAPK signaling pathways, which are able to inhibit CRC cells growth, leading to apoptosis. These promising results may enhance the CRC patients’ survival rate. Moreover, PI3K, AKT, or mTOR inhibitors alone are not very efficient in CRC treatment, because drug resistance appears to be driven by Wnt/β-catenin or by MAPK signaling pathways’ components.

In this context, we conclude that a promising therapeutic strategy for CRC patients may be based on genetic mutation detections and targeting either EGFR/VEGFR in association with PI3K/AKT/mTOR, Wnt/β-catenin, or MAPK inhibitors. This approach could provide new perspectives and new hopes for CRC patients.
